# Long-term assessment of the NHS hybrid closed-loop real-world study on glycaemic outcomes, time-in-range, and quality of life in children and young people with type 1 diabetes

**DOI:** 10.1186/s12916-024-03396-x

**Published:** 2024-04-24

**Authors:** Sze May Ng, Neil P. Wright, Diana Yardley, Fiona Campbell, Tabitha Randell, Nicola Trevelyan, Atrayee Ghatak, Peter C. Hindmarsh

**Affiliations:** 1 Faculty of Health, Social Care and Medicine, Edge Hill University, Ormskirk, UK; 2 Department of Women’s and Children’s Health, University of Liverpool, Liverpool, UK; 3 Paediatric Department, Mersey and West Lancashire Teaching Hospitals, Ormskirk, L39 2AZ UK; 4 Sheffield Children’s Hospital, Sheffield, UK; 5 Children’s Diabetes Team, Oxford University Hospitals NHS Foundation Trust, Oxford, UK; 6 Children’s Diabetes Centre, Leeds Children’s Hospital, Leeds, UK; 7 Department of Paediatric Endocrinology, Nottingham Children’s Hospital, Nottingham, UK; 8 Southampton Children’s Hospital, Southampton, UK; 9 Alder Hey Children’s Hospital, Liverpool, UK; 10 Children and Young People’s Diabetes Service, University College London Hospitals NHS Foundation Trust, London, UK

**Keywords:** Hybrid-close loop, Diabetes, Paediatric

## Abstract

Hybrid closed-loop (HCL) systems seamlessly interface continuous glucose monitoring (CGM) with insulin pumps, employing specialised algorithms and user-initiated automated insulin delivery. This study aimed to assess the efficacy of HCLs at 12 months post-initiation on glycated haemoglobin (HbA1c), time-in-range (TIR), hypoglycaemia frequency, and quality of life measures among children and young people (CYP) with type 1 diabetes mellitus (T1DM) and their caregivers in a real-world setting. Conducted between August 1, 2021, and December 10, 2022, the prospective recruitment took place in eight paediatric diabetes centres across England under the National Health Service England’s (NHSE) HCL pilot real-world study. A cohort of 251 CYP (58% males, mean age 12.3 years) with T1DM participated (89% white, 3% Asian, 4% black, 3% mixed ethnicity, and 1% other). The study utilised three HCL systems: (1) Tandem Control-IQ AP system, which uses the Tandem t:slim X2 insulin pump (Tandem Diabetes Care, San Diego, CA, USA) with the Dexcom G6® CGM (Dexcom, San Diego, CA, USA) sensor; (2) Medtronic MiniMed™ 780G with the Guardian 4 sensor (Medtronic, Northridge, CA, USA); and (3) the CamAPS FX (CamDiab, Cambridge, UK) with the Ypsomed insulin pump (Ypsomed Ltd, Escrick, UK) and Dexcom G6® CGM.

All systems were fully funded by the NHS. Results demonstrated significant improvements in HbA1c (average reduction at 12 months 7 mmol/mol; *P* < 0.001), time-in-range (TIR) (average increase 13.4%; *P* < 0.001), hypoglycaemia frequency (50% reduction), hypoglycaemia fear, and quality of sleep (*P* < 0.001) among CYP over a 12-month period of HCL usage. Additionally, parents and carers experienced improvements in hypoglycaemia fear and quality of sleep after 6 and 12 months of use. In addition to the improvements in glycaemic management, these findings underscore the positive impact of HCL systems on both the well-being of CYP with T1DM and the individuals caring for them.

## What is already known

In the UK, there is a scarcity of real-world published data on the utilisation of hybrid closed-loop (HCL) systems within the paediatric population.

## What this study has found

The NHS England Closed Loop Study in Children and Young People represents a ground-breaking initiative, marking the first nationwide pilot effort to extend universal health coverage for HCL systems. This study stands out as the largest real-world investigation of HCL in the UK, revealing a sustained enhancement in glycaemic management, time-in-range, and quality of life measures. These improvements encompass reduced fear and worry related to hypoglycaemia, as well as enhanced sleep quality for both patients and their caregivers, observed 6 and 12 months post-HCL adoption.

## What are the implications of the study?

This study signals the need for future investigations to delve into the extended impact of HCL in this population over a more prolonged duration. Additionally, it highlights the importance of including hard-to-reach and diverse groups in subsequent research endeavours, ensuring a comprehensive understanding of the potential benefits and challenges associated with HCL systems in paediatric care.

## Introduction

Type 1 diabetes (T1DM) is a chronic condition that requires vigilant management of blood glucose concentrations to prevent both short-term complications, such as hypoglycaemia, and long-term complications, such as cardiovascular disease and kidney failure. Children and young people (CYP) with T1DM face unique challenges, as managing their blood glucose concentrations involves a delicate balance between insulin administration, diet, and physical activity [1]. In recent years, there has been a significant breakthrough in diabetes care with the introduction of hybrid closed-loop systems, offering a promising solution for achieving better blood glucose management while reducing the risk of hypoglycaemia in paediatric patients.

A hybrid closed-loop system (HCL) is an advanced diabetes management technology that automates the delivery of insulin based on real-time continuous glucose monitoring (CGM) [2]. These innovative system aims to bridge the gap between traditional insulin pumps and CGMs, providing a more seamless and dynamic approach to diabetes care. In children and adults with T1D, this technology has demonstrated potential in improving overall glycaemic management and minimising the blood glucose fluctuations that often lead to hypoglycaemic episodes as well as reducing fear of hypoglycaemia [3, 4]. The core components of a hybrid closed-loop system include a subcutaneous CGM, an insulin pump, and a control algorithm. The CGM continuously measures glucose levels in the interstitial fluid, providing real-time data to the pump algorithm. The algorithm then processes this information and adjusts insulin delivery rates accordingly. This closed-loop approach allows for timely and precise insulin adjustments, reducing the reliance on manual interventions from caregivers or the child [5, 6].

In the UK, the National Health Service England (NHSE) commenced a pilot initiative of HCL in CYP across eight paediatric centres. The NHSE Closed Loop Study represents a ground-breaking initiative, marking the first nationwide pilot effort to extend universal health coverage for HCL systems This study reports on the 12-month follow-up of real-world data related to glycaemic management, time-in-range measures, and quality of life impact for CYP and their carers and extends our previously published short-term impact study [7].

## Methods

### Setting

Patients with T1DM were recruited prospectively from the 1st of August 2021 to the 10th of December 2022 under the NHSE real-world HCL observational pilot initiative from eight paediatric diabetes centres in England. Recruitment criteria encompassed individuals under the age of 19 with T1DM who were a minimum of 1 year from diagnosis. Participants were excluded if they were already using a sensor-augmented pump or a HCL system. Additionally, participants needed to have at least one HbA1c measurement before initiating the HCL. Exclusion criteria extended to encompass other medical conditions that could influence glucose metabolism, the use of conflicting devices, and involvement in other ongoing diabetes technology trials or those focused on delaying the onset of T1DM.

Analysis of data on metrics such as HbA1c, time in range (TIR), and frequency of hypoglycaemia was undertaken before the initiation of HCL, as well as at 3, 6, and 12 months post-commencement [8]. Ethnicity information was recorded through the NHS classification system. Various platforms, including Diasend®, Tidepool, Dexcom Clarity™, and the Carelink™ uploader systems, were employed to review HbA1c, TIR, and the percentage of time spent in hypoglycaemic (defined as a tissue glucose concentration of 3.9 mmol/l or less). Children aged 12 and above independently completed the validated Hypoglycaemia Fear Survey (HFS) [9, 10]. Parents of children under 12 completed a modified version known as the HFS-Parent (HFS-P) survey. The HFS-P, a reliable measure adapted from an adult questionnaire, was employed to evaluate fear, anxiety, avoidance behaviours, and worries related to hypoglycaemia in parents and caregivers of younger children with diabetes [11–13]. To assess sleep quality, the Patient-Reported Outcomes Measurement Information System (PROMIS) for Sleep-Related Impairment (SRI) questionnaire was utilised for individuals aged 8 and above, with a modified version completed by parents of those under 8 [14]. The PROMIS-SRI questionnaire evaluates various aspects of sleep disturbance, with higher scores indicating greater disruption. Raw to *T*-score conversions were established based on a large general population sample, and PROMIS item-banks are freely available for both research and clinical applications [15].

The study utilised three HCL systems: (1) Tandem Control-IQ AP system, which uses the Tandem t:slim X2 insulin pump (Tandem Diabetes Care, San Diego, CA, USA) with the Dexcom G6® CGM (Dexcom, San Diego, CA, USA) sensor; (2) Medtronic MiniMed™ 780G with the Guardian 4 sensor (Medtronic, Northridge, CA, USA); and (3) the CamAPS FX (CamDiab, Cambridge, UK) with the Ypsomed insulin pump (Ypsomed Ltd, Escrick, UK) and Dexcom G6® CGM.

### Statistical analysis

The primary outcome was the change in HbA1c over the 12-month period. Secondary outcomes were time in range, frequency of hypoglycaemic events, and the results of sleep and fear of hypoglycaemia questionnaires. The analysis of data was conducted using Statistical Package for Social Sciences 21.0 (version 23; SPSS Inc., Chicago, IL, USA). To ensure the reliability of continuous outcomes, distributions were scrutinised. Results were presented as the mean and standard deviation (SD) for continuous parametric outcomes and the median and interquartile range (IQR) for non-parametric outcomes. For continuous parametric outcomes, Student’s* t*-test was employed, while the Mann–Whitney-Wilcoxon test was used for non-parametric outcomes. Demographic comparisons between participating centres and the impact of the type of pump on HbA1c were assessed using one-way analysis of variance (ANOVA) with Tukey’s honest significant difference (HSD). Only data from patients who completed 3, 6, and 12 months of the study were analysed. A per-protocol approach was chosen for analysis instead of an intention-to-treat approach. This decision was based on the belief that, in real-world scenarios, health care professionals and patients would prefer an effect measure unaffected by adherence levels, unlike the intention-to-treat effect.

Bayesian one-way repeated measure ANOVA was utilised to compare values at the start and 3, 6, and 12 months of the study. Linear regression was employed to explore the relationship between pre-study HbA1c and the change over the 12-month period. To account for regression to the mean, the method of Oldham [16] was used, where change is plotted against the average of the initial and final values, indicating no relationship with a horizontal scatter. This approach was chosen over that of Blomqvist or Yudkin and Stratton due to having only one pre-intervention HbA1c for most patients [17, 18]. A *p*-value equal to or less than 0.01 was considered statistically significant.

#### Ethics

NHS England deemed it unnecessary to seek ethical approval for this study, as it was conducted as part of a service evaluation for CGM and HCL use within the organisations. The study did not impact patient care or management direction, and the collection of data and Quality of Life (QoL) surveys were assessed without altering the course of patient care. All patients were aware and consented to be part of the NHS England study.

## Results

A total of 251 CYP with T1D were recruited, comprising 147 males (58%). The mean age at recruitment was 12.3 years (SD 3.5), ranging from 2 to 19 years. The majority (89%) were of white ethnicity, with 3% Asian, 4% black, and 3% mixed ethnicity. A slight deviation from the 2019 Office for National Statistics estimate was noted, with more individuals from white and fewer from Asian ethnicities. The overall mean duration of diabetes was 6.6 years (SD 3.7), ranging from 1.0 to 15.7 years. The male-to-female ratio was 1.4:1, higher than the typically equal sex ratio for CYP with T1D. Demographic variations between the eight participating centres are detailed in Table 1, with notable distinctions in gender distribution and ethnic makeup in specific centres.

**Table 1:** Recruitment centres and demographics

Centre	Number recruited (*N* = 251)	Gender Male to female (%M)	Age at start of HCL (years)	Duration of T1DM (years)
Southport and Ormskirk Hospital	45	32:13 (71.1)	12.5 ± 3.5	4.3 ± 2.1
Nottingham Hospital	44	24:20 (54.6)	12.4 ± 3.8	6.7 ± 3.8
Alder Hey Children’s Hospital	39	22:17 (56.4)	11.1 ± 4.4	7.2 ± 3.4
Leeds Children’s Hospital	38	17:21 (44.7)	13.0 ± 3.3	5.0 ± 3.0
University College London Hospital	28	13:15 (46.4)	13.1 ± 3.3	7.9 ± 3.8
Oxford University Hospital	24	19:5 (79.2)	13.4 ± 2.8	7.8 ± 3.2
Southampton Hospital	22	13:9 (54.6)	11.7 ± 3.9	7.1 ± 3.6
Sheffield Children’s Hospital	11	7:4 (63.6)	12.1 ± 2.6	5.0 ± 3.0

*HCL*, hybrid closed loop; *T1DM*, type 1 diabetes

Data are shown in years as mean ± standard deviation

Percentage of males at each centre shown in parentheses

Out of the 251 enrolled patients, 239 had complete data at 3 months, 226 at 6 months, and 221 had complete data at 12 months of the study for glycaemic and CGM measures. Non-completion reasons included missed clinic appointments, failure to complete questionnaires, and cessation of HCL due to skin allergies. No baseline biochemical differences were observed between completers and non-completers, except for a shorter duration of diabetes in completers (6.4 years, SD 3.7) compared to non-completers (8.5 years, SD 3.7) (*p* = 0.006). The study utilised three HCL systems: Tandem Control-IQ AP system, Medtronic MiniMed™ 780G, and CamAPS FX. The majority (78%) were on Tandem Control-IQ AP, 11% on CamAPS FX, and 11% on Medtronic 780G. Initial HbA1c values were slightly lower in patients using CamAPS FX but this did not reach statistical significance.

**Fig. 1:**
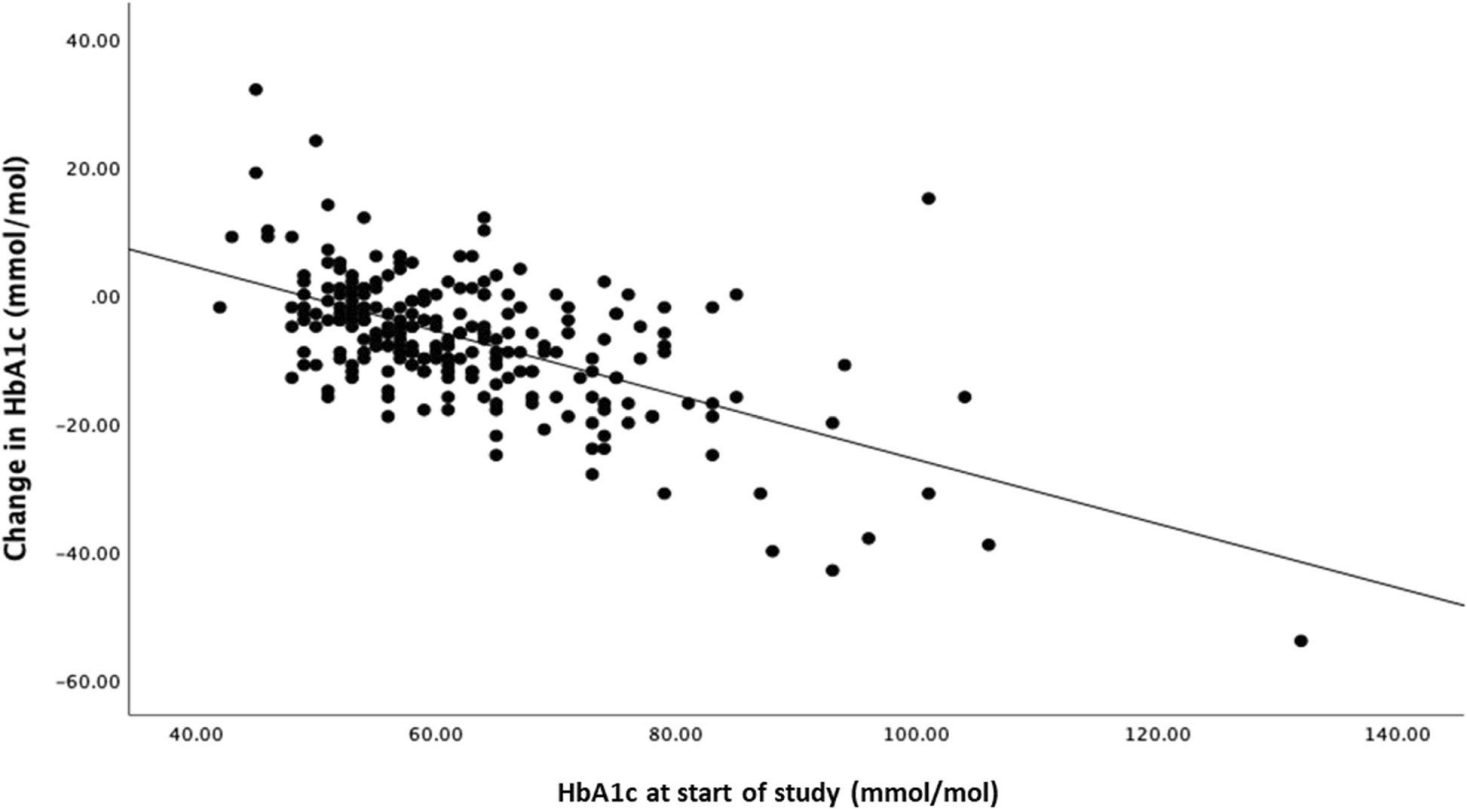
Relationship between glycated haemoglobin (HbA1c) at the start of the study (baseline) and the change in HbA1c at 12 months

Significant improvements were observed in HbA1c, TIR, and frequency of hypoglycaemia after 3, 6, and 12 months of HCL use compared to 3 months prior to starting HCL. HbA1c decreased from 62 (SD 11) mmol/mol at the start to 55 (SD 9) mmol/mol at 6 months and was sustained at 55 (SD 11) mmol/mol at 12 months (*p* < 0.001) (Table 2). A linear relationship was noted between the initial HbA1c and the reduction after 6 and 12 months of HCL use. There was a significant linear relationship (Fig. 1) between the HbA1c at the start of HCL and the reduction after 12 months of HCL use described by the equation: Change in HbA1c at 12 months = -0.50 (Pre HCl HbA1c) + 24.3, *r* = 0.60; *p* < 0.001 (Fig. 1). Using the Oldham approach to testing for regression to the mean a linear relationship between change in HbA1c at 12 months and average of pre- and 12-month HbA1c was observed (*r* =  − 0.21; *p* = 0.001) excluding a regression to the mean as the main explanation for the change observed.

**Table 2:** Comparison of variables pre-HCL vs post-HCL commencement at 3, 6, and 12 months

**Variables**	**Before** **HCL (*n*** **=** **251)**	**3 months after HCL (*n*** **=** **239)**	**Difference (95% confidence interval)**	***P*** **value**
HbA1c (mmol/mol)	62.3 ± 12.1	54.1 ± 7.9	7.7 (6.5 to 8.9)	*P* < 0.001
TIR (%)	48.7 ± 15.3	64.7 ± 11.8	− 15.8 (− 17.6 to − 14.1)	*P* < 0.001
Hypoglycaemia (%)	3.6 ± 3.8	2.4 ± 2.7	1.3 (0.7 to 1.74)	*P* < 0.001
**Variables**	**Before HCL (*n* = 251)**	**6 months after HCL (*n* = 226)**	**Difference (95% confidence interval)**	***P* value**
HbA1c (mmol/mol)	62.3 ± 12.1	55.3 ± 9.3	7.0 (5.8 to 8.2)	*P* < 0.001
TIR (%)	48.7 ± 15.3	63.0 ± 12.4	− 14.3 (− 15.9 to − 12.4)	*P* < 0.001
Hypoglycaemia (%)	3.6 ± 3.8	2.4 ± 2.2	1.2 (0.82 to 1.74)	*P* < 0.001
**Variables**	**Before HCL (*n* = 251)**	**12 months after HCL (*n* = 221)**	**Difference (95% confidence interval)**	***P* value**
HbA1c (mmol/mol)	62.3 ± 12.1	55.4 ± 12.2	6.9 (5.8 to 8.4)	*P* < 0.001
TIR (%)	48.7 ± 15.3	62.0 ± 12.0	− 13.3 (− 15.4 to − 11.4)	*P* < 0.001
Hypoglycaemia (%)	3.6 ± 3.8	2.0 ± 1.6	1.6 (0.9 to 1.9)	*P* < 0.001

*HCL*, hybrid closed loop; *TIR*, time-in-range

Data are shown as mean ± standard deviation

TIR increased to 63% (SD 12) at 6 months and was again sustained at 62.0% (SD 12) at 12 months (*p* < 0.001), while time spent with sensor glucose < 3.9 mmol/l decreased from 3.7% (SD 3.1) to 2.4% (SD 2.2) at 6 months and 2.0% (SD 1.6) at 12 months (*p* < 0.001). A threshold analysis using an HbA1c value of 48.6 mmol/mol (point on linear regression where change in HbA1c was zero) revealed a slight decrease in TIR over the 12-month period and a reduction in the percentage of time hypoglycaemic from 8% pre-HCL to 2.7% after 12 months of HCL use (*P* = 0.02). Fear of hypoglycaemia scores and sleep disturbance scores showed a significant reduction over the 12-month study period (*p* < 0.001) (Table 3). Notably, no differences were observed in biochemical, hypoglycaemia, and sleep score measures after 12 months among the different types of HCL used (Table 4).

**Table 3:** Fear of hypoglycaemia before and after HCL commencement at 6 and 12 months

	**HFS Scores**	**Before HCL**	**6 months after HCL**	**Difference (95% confidence interval**	***P*-value**
Parent/carers	Mean behaviour score	27.0 ± 6.9	22.6 ± 7.6	4.4 (3.1 to 5.7)	*P* < 0.001
	Mean worry score	29.6 ± 12.0	23.1 ± 11.4	6.5 (4.7 to 8.3)	*P* < 0.001
	Mean total score	56.5 ± 16.7	45.2 ± 16.9	11.3 (8.5 to 14.1)	*P* < 0.001
Patients (aged > 12yrs)	Mean behaviour score	31.5 ± 6.0	28.6 ± 6.1	2.9 (1.7 to 4.0)	*P* < 0.001
	Mean worry score	33.7 ± 12.7	29.1 ± 9.7	4.6 (2.7 to 6.5)	*P* < 0.001
	Mean total score	64.9 ± 15.3	57.5 ± 12.7	7.4 (4.8 to 9.9)	*P* < 0.001
Parent/carers	**HFS scores**	**Before HCL**	**12 months after HCL**	**Difference (95% confidence interval**	***P*-value**
	Mean behaviour score	27.0 ± 6.9	20.9 ± 7.5	6.1 (4.5 to 8.2)	*P* = 0.048
	Mean worry score	29.6 ± 12.0	21.4 ± 11.0	8.2 (6.8 to 11.9)	*P* = 0.005
	Mean total score	56.5 ± 16.7	42.2 ± 16.0	14.3 (12.3 to 19.5)	*P* < 0.001
Patients (aged > 12yrs)	Mean behaviour score	31.5 ± 6.0	28.5 ± 7.4	3.0 (1.2 to 4.6)	*P* = 0.002
	Mean worry score	33.7 ± 12.7	27.6 ± 10.1	6.1 (3.4 to 9.3)	*P* < 0.001
	Mean total score	64.9 ± 15.3	57.7 ± 14.6	7.2 (3.9 to 11.0)	*P* < 0.001

*HCL*, hybrid closed loop; *HFS*, hypoglycaemia fear score

Data are shown as mean ± standard deviation

**Table 4:** Sleep *T*-scores before and after HCL commencement at 6 and 12 months

	**PROMIS scores**	**Before HCL**	**6 months after HCL**	**Difference (95% confidence interval**	***P*-value**
Patients (aged > 8 years)	PROMIS-Sleep-Related Impairment *T*-score	56.6 ± 9.1	54.9 ± 9.3	1.7 (0.3 to 3.0)	*P* = 0.017
Parent/carers	PROMIS-Parent Proxy Sleep Disturbance T-score	60.1 ± 10.4	56.1 ± 10.5	4.0 (2.2 to 5.6)	*P* < 0.001
	**PROMIS scores**	**Before HCL**	**12 months after HCL**	**Difference (95% confidence interval**	***P*-value**
Patients (aged > 8 years)	PROMIS-Sleep-Related Impairment T-score	56.6 ± 9.1	53.1 ± 10.8	3.5 (1.9 to 5.8)	*P* < 0.001
Parent/carers	PROMIS-Parent Proxy Sleep Disturbance T-score	60.1 ± 10.4	54.1 ± 10.5	6.0 (3.1 to 7.9)	*P* = 0.002

*HCL*, hybrid closed loop; *PROMIS*, Patient-Reported Outcomes Measurement Information System; data are shown as mean ± standard deviation

## Discussion

The NHSE Closed Loop Study demonstrated notable and sustained improvements in various diabetes-related parameters over a 12-month period. These enhancements included better glycaemic management, increased TIR, reduced frequency of hypoglycaemia, diminished hypoglycaemia fear, and improved quality of sleep for CYP. These findings align with existing real-world data, suggesting the superiority of HCL systems in achieving target glucose range, preventing hypoglycaemia, and reducing glycaemic variability. The positive impact extended to parents and carers, who also experienced improved hypoglycaemia fear and sleep quality at 12 months. Notably, the observed changes in glycaemic measures, TIR, and hypoglycaemia frequency were consistent at 3, 6, and 12 months suggesting the absence of a Hawthorne effect during this period. The improvements in hypoglycaemia fear and sleep quality represent significant advancements, relieving individuals of the burdens associated with diabetes. To put the HbA1c data in perspective, the baseline HbA1c (62 mmol/mol) was similar to that recorded in the National Paediatric Diabetes Audit (NPDA) 2021/22 (61 mmol/mol) and was reduced by 7 mmol/mol during the course of the study whereas the NPDA value had remained unchanged over the previous audit year.

One of the primary advantages of HCL systems is their ability to maintain blood glucose concentrations within a target range more consistently [19, 20]. This is particularly crucial in children, as their glucose concentrations tend to fluctuate more rapidly due to factors such as growth, physical activity, and varying meal sizes. The HCL system adapts to these changes in real-time, leading to improved overall glycaemic management. Hypoglycaemia is a constant concern for caregivers of children with T1DM. The HCL system’s ability to detect and respond to falling tissue glucose concentrations helps prevent hypoglycaemic episodes. By providing precise insulin delivery adjustments, the system minimises the risk of overshooting and causing low blood glucose concentrations, a common concern with manual insulin administration.

Nocturnal hypoglycaemia is a significant concern for parents of CYP with T1DM. Hybrid closed-loop systems have demonstrated efficacy in maintaining stable overnight glucose concentrations, providing parents with greater peace of mind and ensuring that children can enjoy uninterrupted sleep. Numerous clinical trials have investigated the efficacy of HCL systems in CYP with T1DM, consistently showing positive outcomes [21, 22].

The landmark “International Diabetes Closed-Loop” (iDCL) trial, for instance, demonstrated that CYP using HCL systems experienced a significant reduction in time spent in hypoglycaemia compared to those using conventional pump therapy[23]. Additionally, a study published in the New England Journal of Medicine found that CYP aged 6 to 13 years using a HCL system achieved better glycaemic management and spent more time in the target glucose range compared to those using a traditional insulin pump [24]. These findings underscore the potential of HCL systems to revolutionise diabetes management in the CYP population [25]. While the benefits of HCL systems in paediatric diabetes care are evident, challenges and considerations remain. Issues such as system accuracy, device adherence, and the need for periodic sensor calibrations may pose practical challenges for some families. Moreover, the cost of these systems and the accessibility to the latest technology can be barriers for widespread adoption.

While the real-world study drew data from an unselected diabetes population, providing strength in its large and representative sample, the predominantly white population raises considerations about diversity as a limitation of the study. Another limitation was the intention to treat was not used as analyses as detailed information for dropout was not available within a real-world study. The linear relationship between the decrease in HbA1c during HCL use and the starting value, while accounting for regression to the mean, suggests potential benefits for individuals struggling with higher HbA1c values. Furthermore, the study implies that even patients with initially optimal HbA1c values can benefit further from HCL systems, particularly in terms of reducing time spent in hypoglycaemia which improved over the 12-month study period. The study was not constructed to determine whether any one HCL system was superior to another, so usage at present will be determined by the patient choice of the options available. The reduction in hypoglycaemia fear and improved sleep quality, likely attributed to reduced time spent with low tissue glucose, contribute to a decrease in overall diabetes burden. These positive outcomes were shown to be sustained and had a longer assessment period than many clinical trials that do not go beyond 6 months. It further underscores the potential role of HCL in managing diabetes in CYP, warranting further exploration of its cost-effectiveness and overall impact on diabetes burden.

## Conclusion

Hybrid closed-loop systems represent a ground-breaking advancement in the management of T1DM in CYP, offering a more automated and responsive approach to insulin delivery. The evidence from clinical trials consistently supports the use of these systems in achieving better glycaemic management and reducing the risk of hypoglycaemia. This study demonstrates the sustained improvements over a 12-month period in a real-world setting which was comparable to other real-world studies[26]. As technology continues to evolve, addressing challenges related to accuracy, accessibility, and cost will be crucial in ensuring that these innovative solutions become standard paediatric diabetes care. Ultimately, the integration of HCL systems has the potential to transform the lives of CYP with T1DM, providing them with greater freedom, improved health outcomes, and a brighter future.

## Data Availability

Available on request.
